# Conceptual Model for Integrating Environmental Impact in Managerial Accounting Information Systems

**DOI:** 10.3390/ijerph18041791

**Published:** 2021-02-12

**Authors:** Sorina Geanina Stanescu, Ion Cucui, Constantin Aurelian Ionescu, Liliana Paschia, Mihaela Denisa Coman, Nicoleta Luminiţa Gudanescu Nicolau, Marilena Carmen Uzlau, Mihaela Leasa Lixandru

**Affiliations:** 1 Institute of Multidisciplinary Research for Science and Technology, Valahia University of Targoviste, 130004 Targoviste, Romania; geaninastanescu@yahoo.com; 2 Doctoral School of Economics and Humanities, Valahia University of Targoviste, 130105 Targoviste, Romania; ioncucui50@gmail.com (I.C.); mihaelalixandru89@yahoo.com (M.L.L.); 3 Academy of Romanian Scientists, 50044 Bucharest, Romania; 4 Faculty of Economics, Hyperion University of Bucharest, 030615 Bucharest, Romania; paschialiliana@gmail.com (L.P.); n.gudanescu@gmail.com (N.L.G.N.); carmen.uzlau1812@gmail.com (M.C.U.); 5 Institute of National Economy, Romanian Academy, 050711 Bucharest, Romania; 6 Institute of Economic Forecasting, Romanian Academy, 050711 Bucharest, Romania

**Keywords:** management accounting, EMA, environmental costs, environmental impact, conceptual model

## Abstract

The main research objective is to develop a conceptual accounting model to reflect the environmental impact generated by the economic activity of Romania’s entities. In order to identify the current stage of the use of environmental accounting by the Romanian economic entities, the questionnaire used was based on a random sample of 377 entities whose economic activity has a significant impact on the environment. The results suggest the need to develop a model for integrating environmental impact into accounting. The model is based on the description of the technological process and determination of the environmental impact on each activity, stage, or procedure of the technological process, which enterprise will integrate its monetary value in the cost of production and will reflect it in the management accounting system, using specific environmental accounting instruments. The model involves five stages, and by combining internal and external information provided by environmental management accounting is a relevant source for substantiating decisions to promote environmental responsibility in Romanian companies.

## 1. Introduction

Environmental accounting has become a concern for more and more organizations, due to the need to identify environmental issues and assess environmental performance [1,2,3,4]. In the early 1970s, Bertrand de Jouvenel was the first author who investigated a traditional critical accounting method, taking care in only commercial goods and services for the market, without being interested in effects on nature [5]. Environmental accounting has developed as a means of management and control of economic entities to meet their immediate needs, evolving rapidly as a means of information for both the management of economic entities and for employees, or for third parties in general [6,7]. Thus, the use of environmental accounting, and the implementation of environmental protection strategies, can be a competitive advantage for many organizations following the principles of sustainable development and social responsibility [8,9]. Environmental accounting involves efficiently reflecting the environmental impact, based on correlating the information provided by management accounting with non-financial ones, calculating costs, and reporting environmental information to potential users. Environmental accounting’s central role is to address environmental issues that impact sustainable development goals and influence economic entities’ behavior by promoting social and environmental responsibility [10,11]. 

Environmental accounting is a tool that can be used to monitor and reflect economic activities related to the environment. Several specialists have researched this field according to various topics, including sustainable development accounting, environmental, financial accounting and, environmental management accounting [12,13,14,15,16]. Some discussed the impact of socio-economic activities on the environment [17], the challenges facing environmental accounting to respond to sustainable development strategies’ needs, and lay the foundations of ecological civilizations [18,19,20,21]. Others focused more on reporting environmental information and evaluating environmental performance; several researchers have highlighted the need to develop a model for implementing environmental accounting [22,23]. The results of previous studies indicate that there are no standard models for implementing environmental accounting. Nevertheless, Lamberton Geoff (2005) takes an essential step towards formalizing an accounting model of sustainability, establishing the following main components: objectives; principles; techniques for collecting, recording, and evaluating data; reports; and qualitative characteristics of information [24]. Patrick de Beer and Francois Friend published the Environmental Engineering Group environmental costing model (EEGECOST) to promote environmental accounting in South Africa [25]. Jacques Richard presented an accounting model adapted by involving the environment: “La Comptabilité Adaptée au Renouvlement de l’Environnement” (CARE) [26]. At the national level, environmental accounting had undergone a comprehensive development since 2008, when Romania became a European Union member. Thus the Romanian economic entities had to comply with European standards and requirements. Ienciu Ionel (2009) considers that, to reflect a real and correct image of the company’s impact on the environment, it requires two pillars that complement the traditional accounting: the environmental management accounting, and the financial reports that give the users a clear and comprehensive for an overview of the environmental performance [27]. Based on these recommendations, the main research objective is to develop a useful conceptual accounting model to reflect the impact of environmental accounting for Romania’s economic entities to promote sustainable development and economic growth.

The theoretical and empirical research results based on multiple institutional and practical approaches have highlighted the need for a conceptual model for implementing environmental management accounting (CM-EMA). Thus, the CM-EMA model was developed as a fundamental support for Romanian companies to develop and implement environmental accounting. The CM-EMA model is based on identifying the environmental impact exerted by the economic activity and its internalization by integrating the environmental costs into the total cost of the company’s products, services, or works. The model involves five stages: 1. Identification of significant influence that the company has on the environment; 2. Determination of impacts exerted by economic activity on the environment; 3. Identifying and allocating environmental costs caused by environmental impact; 4. Internalization of externalities—the integration of environmental costs in production; 5. Identification of the environmental information provided by environmental accounting and their use for achieving environmental reporting or forecasting. This model aims to provide a conceptual framework and a practical methodology for assessing the environmental impact of the economic entity’s economic activity. This accounting model aims to supplement financial accounting with the information provided by environmental accounting, which helps to reflect environmental costs and helps identify and record environmental revenues or benefits and report this information to the enterprise’s internal and external users. The research results are relevant to the economic environment and the academic environment, primarily through the developed accounting model. The analysis of the results obtained based on the submitted questionnaires and the strategic interpretations helps understand how the Romanian economic entities reacted to the legislative and institutional requirements regarding sustainable development and social responsibility.

## 2. Literature Review

The mobilization of economic entities in environmental protection activities resulted from an awareness process in the 1950s, when the industrial society was booming [27,28]. Awareness of ecological problems involves harmonizing economic and social development with the rational and prudent management of non-renewable resources [29,30]. At the global level, the first debate on environmental protection was organized in June 1972, at the United Nations World Conference in Stockholm [31]. Interest increased with the Rio de Janeiro Earth Summit organization in 1992, when Agenda 21 is adopted, and the term “environmental accounting” is introduced as an essential tool for sustainable development, necessary for the implementation of a coherent environmental protection policy. There were proposed the internalization of environmental costs and economic instruments for environmental impact assessment, and all these actions were based on promoting the “polluter pays” principle [32,33]. Environmental accounting can be defined as a set of organizational activities for measuring and evaluating environmental performance and reporting these results, covering all areas of accounting that may be affected by the effects of environmental problems, including new areas of eco-accounting [34].

The emergence and development of environmental management accounting (EMA) have offered new organizational management approaches and tools. Bennett Martin considers environmental management accounting “the link between environmental management and management accounting” [35]. Environmental management accounting is a combined approach that provides financial and accounting information, represented by the costs and balance sheets of material flows (Table 1). It improves the efficiency of materials use by reducing risk and environmental impact, thus reducing environmental protection costs [36]. Environmental accounting has established itself as a necessary tool in substantiating decisions, a system used to organize, manage, and provide data and information on the environment in physical or monetary units [17,37]. It presents an objective view of the status and changes of natural heritage, the interactions between the economy and environment, and expenditure on prevention, environmental protection, and repairing environmental damage [38]. Environmental accounting is a system for planning and managing environmental performance, reflecting the entity’s management’s managerial style and commitment towards the environment [39,40]. Environmental accounting is an essential element for evaluating, identifying, analyzing, and recording the impact produced by the economic activity on the environment and using this information in the financial and decision-making process [41]. Therefore, environmental accounting is a logical extension of traditional accounting, which considers the current reality, representing an indispensable management tool for sustainable development [42]. Economic activities can negatively affect the entities’ production, costs, and profit through the impact on the environment and consumer welfare through changes they cause in the market. Thus, reconsidering the financial accounting system by integrating environmental issues is no longer an option but a way forward by all national and international companies [41]. 

Standardization of the concepts of environmental accounting (EA) and environmental management accounting (EMA) has been achieved by the International Federation of Accountants (IFAC). IFAC defines environmental management accounting as managing economic and environmental performance by developing and implementing appropriate systems and practices regarding the environment [43]. Although this also includes reporting and auditing activities in some organizations, EMA typically refers to lifetime costs, total costs, benefits assessment, and strategic environmental management planning [44,45]. Decision-makers within an organization can use the information provided by environmental management accounting to make decisions that impact both the organization’s financial performance and the environment [46,47,48,49]. Identifying the environmental costs related to a product, work, service, or economic system, and recognizing the entity’s accounting is necessary to adopt optimal managerial decisions [50,51,52].

Like the other European countries and Romania, there are significant problems regarding the protection of the environment. While traditional financial accounting only reflects in the annual financial statements the stocks and flows of an entity from an economic point of view, environmental accounting presents additional information under the time, place, and type of impact [51,53]. Implementing an environmental accounting system in economic entities brings benefits that can vary considerably depending on their specific activities [47,54,55,56]. Until a few years ago, non-financial information reporting was not mandatory, being left to each economic entity’s discretion. The Directive 2014/95/EU was transposed at the national level by Order of the Ministry of Public Finance 1938/2016. Starting with 2018, entities that exceed the average number of 500 employees must include a non-financial statement in the directors’ report. This contains the financial information on the performance, position, and impact of the activity and non-financial information regarding the environmental, social, and personnel aspects, the respect of human rights, the fight against corruption, and bribery. Romanian entities are increasingly using non-financial reporting, including social, environmental, and economic impact, as a tool for responsibility and a tool for designing the company’s strategy and discovering new income and sustainability [57,58]. Depending on the non-financial information presented in the annual statements, Romanian entities can be classified as follows: entities that responsibly have environmental information, and a detailed report, entities that summarize environmental information, and companies that do not take into account environmental issues and do not present this information in any report [59]. 

Researchers at the national level have shown that implementing the environmental management system within the Romanian entities can resolve conflicts between the economy and the environment and contribute to the increase in economic performance [60]. For an accurate image of the entities’ environmental impact, the entity must have an efficient accounting system that integrates the environmental aspects in the decision-making and financial process [61,62].

Industrial costs for environmental protection, including pollution reduction, waste management, monitoring, reporting, legal fees, and insurance, have increased rapidly over the past 30 years, in line with increasingly stringent environmental regulations [63]. Accounting systems traditionally attribute many of these environmental costs to general costs (which are not directly allocated), this having the consequence that the product managers are not stimulated to reduce the environmental costs and are not directly aware of the magnitude of these costs [64,65]. When environmental costs are considered general, product lines are divided, products with low costs subsidize those with high costs, leading to incorrect product pricing, which reduces profitability [66,67]. By identifying, evaluating, and allocating environmental costs, management is allowed to identify cost-saving opportunities and create a clean production system that applies to the entire production cycle for: increasing productivity by ensuring more efficient use of raw materials, energy, and water; promoting superior performance by reducing, at source, waste, and emissions; reducing the impact of products on the environment, during their life cycle, by designing environmentally friendly, cost-effective products [68,69,70,71,72].

## 3. Methodology

Starting from the idea that economic entities should have a behavior-oriented towards social responsibility, and the integration of environmental aspects in the managerial accounting system is an absolute necessity, the following hypotheses were formulated:

**Hypothesis** **1 (H1).** *The activities specific to sustainable development and social responsibility are influenced by the sphere of action, the form of ownership, the entity’s size, and the advantages and disadvantages that it could obtain/encounter in this process;*

**Hypothesis** **2 (H2).** *There are associations between the environmental impact exerted by the economic activity and the integration of the specific instruments of social responsibility at the financial entities level;*

**Hypothesis** **3 (H3).** *There is a correlation between identifying, integrating, and quantifying environmental costs and developing environmental management accounting;*

**Hypothesis** **4 (H4).** *The integration of environmental impact in accounting is possible by creating a model for reflecting the environmental impact of the management accounting information system.*

The research hypotheses were validated through deductive reasoning and using the statistical research tool based on the questionnaire. The questionnaire elaborated was structured in three sections. Section I is intended for the general profile of the economic entity and aims to obtain information regarding the field of activity, the size of the entity, as well as information regarding the market and the products developed by it. This section contains five closed questions, one of which is based on the Likert scale. In Section II the sustainable development and social responsibility of the entity are evaluated by seven closed questions by which the respondents express their opinion on the following aspects: knowledge of the legislation in the field of environmental, the interest of the economic entity regarding the environmental management systems, and the environmental impact assessment. In Section III, the use of environmental accounting aims to obtain information regarding the integration of the environmental impact into the accounting system, the environmental costs and expenses recorded by the company and the share of these costs by nine items.

The study’s target group is represented by Romania’s economic entities that significantly impact the environment in the fields: forestry, extractive industry, processing industry, energy industry, transport, and construction. These areas were chosen following the analysis of the report “Expenditure, production, and financing of environmental protection in 2018”, a report provided by the National Institute of Statistics. The economic entities in the mentioned fields registered the highest share of the expenses on environmental protection. The questionnaire was sent to 377 respondents targeted by e-mail in October 2019 (first request), followed by a second request in November 2019, obtaining the following results: 302 questionnaires received, 288 valid questionnaires, 14 invalid questionnaires, 76.39% rate of response. Respondents were both management staff and operational staff working in the financial-accounting department of the economic entities participating in the study. The statistical interpretation and analysis of the data collected from the questionnaire and the graphical representations were made using the specialized application SPSS Statistics 21. The reliability analysis and the questionnaire’s constructive validation were performed by analyzing the internal consistency of its items with the Alpha Cronbach coefficient’s help, both for the total scale and each section (Table 2). The results of the study show an alpha coefficient of 0.970. The analysis on each section showed a variation of the coefficient between 0.873 and 0.967, demonstrating a strong intercorrelation between chosen intimates.

According to the literature, these values show a moderate to strong correlation between the areas analyzed for the final version of the questionnaire [73,74]. The element-domain correlation analysis showed mostly positive and significant coefficients from moderate to strong, except for questions Q4 (0.200), Q8 (0.324), Q9 (0.472), and Q15 (0.435), which have values less than 0.500, which means that they are not significantly correlated. However, still, the value of the total coefficient would not change significantly if the analysis of these answers were abandoned. For each item, frequencies and percentages were calculated, and statistical methods and tools were used to validate research hypotheses, such as Chi2 test, Likert scale, Kendall’s, cross-analysis-Crosstable, Cramer’s V statistical index, Pearson correlation, Spearman’s rank correlation index. For the final section of the questionnaire, a factorial analysis of environmental accounting was performed, using the Correlation Matrix.

## 4. Results

**Figure 1:**
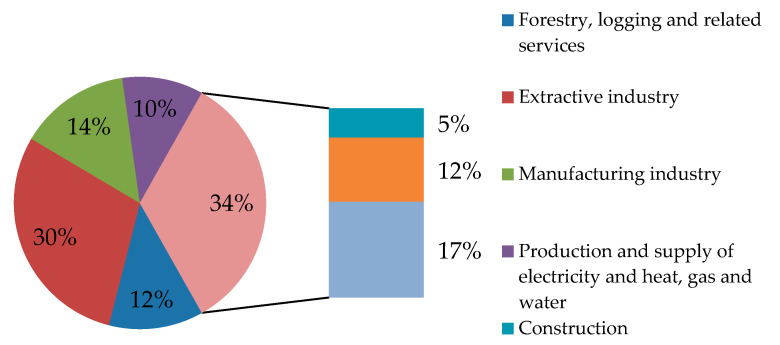
Distribution of the questioned entities by activity areas.

The sphere of activity of the participating respondents are hierarchized and presented in Table 3 and Figure 1, economic entities from various fields being analyzed, observing, 85 validated answers (30%) for the extractive industry, 49 (17%) for the public administration, and 41 (14%) for the processing industry. The construction companies gave the lowest weight, with only 15 (5%) of validated questionnaires. The Chi2 test shows that the empirical distribution of the sample is significantly different from the theoretical distribution. In this respect, the heterogeneity of companies’ distribution, according to the seven activity fields, is statistically significant.

**Figure 2:**
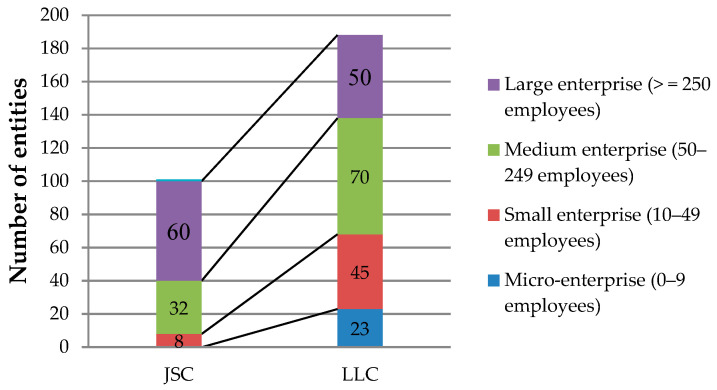
Classification of economic entities.

The classification of the economic entities participating in the study, according to the form of ownership is divided into two main categories: 100 joint stock companies (JSC), (35%) and 188 limited liability companies (LLC), (65%). Figure 2 shows the participating economic entities according to their size and according to the number of employees.

Regarding other characteristics of identification of the companies participating in the study, the descriptive analysis of variables, such as the target market, the selling price of the products, the quality of the products, the image on the market of the products, the bio characteristics of the products, the involvement in ecological investments, and the policies and environmental strategies, were calculated by evaluating the average level of participation and by assessing the dispersion of responses for each domain by calculating the standard deviation (Table 4).

**Figure 3:**
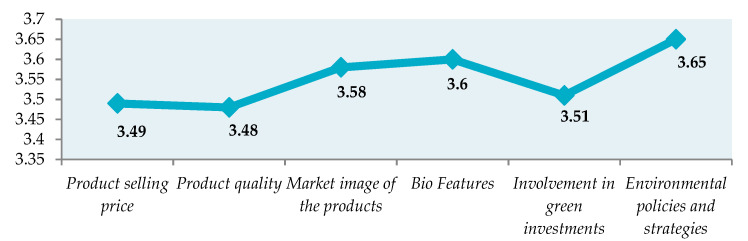
The characteristics of the economic entity analyzed in comparison with the main competitors.

It can be observed that the standard deviation is lower for the target market variable (0.601). For the other variables, the standard deviation is between 0.918 and 1.026, which demonstrates homogeneity in the validated responses. Using the Likert method, the product sales price level, product quality, product-market image, bioproduct characteristics, economic entity involvement in ecological investments, and environmental policies and strategies of the economic entities participating in the study, compared to the main market competitors, were studied (Table 5). The Likert scale allows respondents to have more answers. To measure their attitudes, scores are assigned to different responses. There were awarded scores between 1 and 5 (the classic version of Likert—in five stages). In essence, 1 represents far below the level of the competitors, 2 under the level of the competitors, 3 at the same level as the main competitors, 4 above the level of the competitors, and 5 far above the level of the competitors. The “I don’t know” answer was eliminated to encourage them to make their choices known, and to avoid biasing the responses (Figure 3). 

All six characteristics analyzed in the economic entities included in the study are at the same level or slightly above the values of the main competitors. The characteristics that stand out from the main competitors’ level are “Bioproduct characteristics” and “Environmental policies and strategies” developed by the economic entity.

The second section of the questionnaire focused on obtaining relevant information on the actions taken by the economic entities participating in the study in sustainable development and social responsibility. The respondents express their opinion on the degree of commitment of the economic entity of which it is a part of the implementation of the practices of social responsibility and sustainable development. In this section, the questions release how these tools are known and used by the companies participating in the study (Table 6).

Thus, these tools are known at the companies’ level, and over 50% of these companies use or have these tools in the implementation. Among the activities developed in the field of social responsibility and sustainable development by the analyzed entities, it was found improvement of working relationships with employees (19.80%), environmental management and material recycling (14.90%), compliance with social working conditions (10.10%), implementation of a code of ethics and deontology (9.40%), environmental impact monitoring, correspondence with international standards, and use of renewable energy sources (6.90%). Among the less implemented activities are ecological transport (1.70%), and the use of non-polluting materials (1.40%). Among the reasons that determined the economic entities to adopt these actions, it can be mentioned: the conviction and the civic involvement (32.60%), the compliance with the regulations, especially at the environmental level (30.20%), the prevention of risks (16.00%), and the requirements imposed at national and international level (12.20%). The economic and competitive advantages, respectively, the image of the entity, are also mentioned by the analyzed entities, in a small percentage of responses (5.90% and 3.10%). Regarding the obstacles that the entities encountered in implementing sustainable development, most respondents consider the employees’ lack of interest as the main disadvantage (25.00%). Additionally, the lack of financial means (20.80%), the lack of interest of customers or suppliers (20.10%), the lack of a development strategy (20.50%), or the lack of time (11.10%) were other variants of response.

An essential role in analyzing environmental problems is the impact exerted by the economic activity on the environment. Overall, 90.30% of the respondents consider that economic activity impacts the environment, and only 9.70% think that economic activity has no impact on the environment. Regarding the assessment and integration of the environmental impact in the financial and managerial system, 93.10% replied that they are interested in evaluating the environmental impact produced by the economic entity’s activity, and only 6.90% answered negatively to this question.

The last section of the questionnaire looks at issues related to integrating the environmental impact by using the information provided by environmental accounting in the economic entities included in the study. The integration of the environment in the financial and managerial system can be achieved by identifying and evaluating from a monetary perspective the impact on the environment (34.70%), by determining the environmental costs (24.30%), by integrating the environmental costs in the decision-making process (23.60%) and by the non-financial reporting (17.40%). Among the obstacles that stand in the way of this process, 48.60% of the respondents believe that the lack of an accounting model and 39.60% consider that the lack of a clear methodology is the main drawbacks (Table 7).

At the national level, expenditures on environmental protection were about 14.70 billion lei, representing 1.6% of GDP, and the share of the most significant expenditures was recorded in the field of waste management [75]. As a result, it is essential to note in which area the entities participating in the study registered the highest share of environmental costs. Even for the entities participating in the study, the largest share is held by the expenses on waste management 51.40%, followed by the expenses on wastewater management 19.10%, and the other types of expenses (expenses for air protection, expenses for soil and groundwater protection), expenses for the protection of biodiversity and landscape, expenses for the reduction in noise and vibration, expenses for the protection against radiation, expenses for the research and development in the field of environmental protection and other environmental activities) record weights much below 10%. Of the specific management accounting methods used to integrate environmental information into the accounting system, most respondents consider the Target Costing method to be the most appropriate for use (102 (35.40%)), followed by the Activity-Based Costing (ABC) method, a method validated by 91 (31.60%) of the respondents. The advanced methods of calculating costs are much more appreciated than the traditional methods, which ranked the last two in the study participants’ preferences.

H1 Validation: The activities specific to sustainable development and social responsibility are influenced by the sphere of action, the form of ownership, the entity’s size, and the advantages and disadvantages that it could obtain/encounter in this process.

By applying the Kendall statistical test (Table 8), it can be observed that the activity fields, the form of ownership, the size of the company, the target market, the legislation in the field of environmental protection, the actions are taken as well as the obstacles that the analyzed companies can face have a strong dependency relationship.

In recent years, the importance of environmental issues for the industry has increased exponentially, in conjunction with the field’s legislative policies. Increasing consumers’ awareness about the impact on the environment has created new demands and opportunities for companies. Additionally, the pressure of NGOs, and increasingly restrictive policies aimed at amending the environmentally harmful behavior, has encouraged companies to control the effects of their activities, to reduce the impact on the environment and avoid additional costs. The environmental impact assessment and quantification have become important strategic tools to achieve sustainable development in national industries in response to increasing environmental legislative pressures. Despite its importance and benefits, companies’ participation in the development of environmentally friendly products and the implementation of a more environmentally friendly production process remains unsatisfactory.

H2 Validation: There are associations between the environmental impact exerted by the economic activity and the integration of the specific instruments of social responsibility at the financial entity level. To obtain a relevant result to validate this hypothesis, a cross-sectional analysis (Crosstable) is required by which the variable “environmental impact” is analyzed against the variable “environmental management” (Table 9 and Table 10).

The statistical index Cramer’s V demonstrates the existence of a link with an intensity of 62.9%, which implies an over-dependence between the environmental impact and the environmental management system, thus validating research Hypothesis 2.

The Pearson coefficient analysis was applied to test the link between the variable “environmental impact” and the tools specific to sustainable development. The correlation between the dependent variable “environmental impact” and the independent variables “environmental management system/environmental performance assessment/quality system/environmental policy development/environmental budgeting/reporting non-financial information” is represented in Table 11. The Pearson coefficient value is positive and relatively high (0.685–0.911), which shows a very high dependency relationship between these variables and significance of <0.05, demonstrates the connection of a directly proportional relationship of very high intensity between the level of environmental impact and the use of specific tools for environmental protection. 

H3 Validation: There is a correlation between identifying, integrating, and quantifying environmental costs and developing environmental management accounting at the level of the analyzed entities.

The cross-sectional analysis of the variables “the extent to which the economic activity generates information on environmental costs”, “annual value of the environmental costs”, and their weight in the total of the expenses recorded by the economic entity was realized by applying the Pearson coefficient, the results of which are presented in Table 12. Between the annual value of the environmental costs and the extent to which the economic entity generates information on the environmental costs, there is a very close dependency relation, the Pearson coefficient registering the value of 87.00%. 

However, the correlation between the share of environmental costs and their value is insignificant, as well as that between the share of environmental costs and the extent to which the activity generates information on environmental costs. 

The detailed analysis of the answers continues with a contingency table (Table 13) in which the opinions of the respondents regarding the benefits brought by the integration of the environment in the accounting system and the integration of the environmental costs in the pricing strategy are highlighted. This analysis shows that the respondents consider a benefit the integration of the environment in the accounting system. The integration of the environmental costs in the strategy of price substantiation is achieved to a large extent for 140 of the respondents. 

The analysis of the correlation of the two qualitative variables (Table 14) shows a positive correlation (0.485), doubled by a confidence coefficient of 0.000, which shows that the directions of action targeted by the strategy of price pricing by including the environmental costs are appreciated in a vast number of respondents.

This information leads to the validation of the hypothesis regarding correlations between the identification, integration, and quantification of environmental costs and the development of environmental accounting at the analyzed companies’ level.

H4 Validation: The integration of the environmental impact in accounting is possible by developing a model for reflecting the environmental impact in the management accounting information system.

The study of the potential obstacles that affect or could affect the company’s ability to use the specific tools of environmental accounting and the integration of the environmental impact is essential, as the current discussions regarding this specific context in the literature are still limited. Additionally, the national industries, especially the Romanian production industries, are in a continuous development and innovation process, and they can harm the environment. Understanding the obstacles to integrate environmental aspects into the financial and decision-making system is very important from the decision-makers’ innovation policy. First of all, it is essential to know the management’s attitude regarding integrating the environmental impact, and secondly, it is essential to identify the barriers frequently encountered by the companies that engage in this process.

In order to validate this hypothesis, it is necessary to establish whether there is any connection between the process of integrating the environmental impact into the managerial system of the company and the main disadvantage reported by the study respondents (lack of a conceptual accounting model), thus, also based on the correlation matrix, by factorial analysis of the items analyzed in section III of the questionnaire (Table 15).

Examination of the Correlation Matrix reveals relatively high correlations between the 10 variables written to measure the tools used by Romania’s entities concerning environmental accounting. For example, the intercorrelations between the variables of barriers integrated environmental impact, environmental expenses recorded, annual value, and top management attitude are greater than 0.80. Similarly, the intercorrelations between environmental cost strategy, generated environmental cost, environmental expenses recorded, annual value, and optimal method is also greater than 0.50. Given the number of high intercorrelations between environmental accounting instruments, the hypothesized factor model appears appropriate.

Applying statistical tests on the data collected through questionnaires is a sure way to characterize the statistical population. Based on the information presented, it can observe a strong connection between integrating the environmental impact into the company’s managerial system and the lack of a conceptual accounting model that could facilitate this process. Thus, consider it appropriate and even necessary to develop and implement an accounting model to integrate the environmental impact in accounting, especially for companies whose economic activity significantly impacts the environment.

## 5. Discussions

A model for reflecting in the accounting managerial information system of the environmental impact implies the permanent optimization of the technological processes through the participation of all the stakeholders in the economic entity and also a collaboration and transparency in the process of informing and evaluating the entire procedural course, which involves a language and an overview in common actions to achieve the transfer of experienced knowledge, the intensive involvement of the leaders of all the managerial structures of the economic entities, the use of the technical means for calculating and estimating the costs, by including a value engineering.

The structure of CM-EMA consists of five stages. We define the model as a set of concepts, principles, and procedures, rules that determine the methods for recognizing, recording, measuring, and reporting financial and environmental transactions.

Stage 1 (Identifying significant influence that the company has on the environment). The economic activity involves different influences on the environment, the demand for raw materials, energy, intermediate goods, the flow of pollutants and waste, and natural spaces employment sites. These influences are mitigated by systems and mechanisms of absorption (waste assimilation, absorption of pollution) and environmental regeneration procedures. If the pressure is higher than the absorptive capacity, they cause changes in the environment, leading to its degradation. Traditionally, the ultimate goal of a business is to maximize profit. However, business objectives revolve around three main axes: to exist, make profits, and grow. Thereby, the commitment to consider environmental issues depends on the objectives set by the company. Economic activity can affect the production, costs, and profits of other entities through the effects they cause on the environment. At this stage, aspects of the company’s location will be investigated carefully, in terms of its interaction with the environment, to identify sources of high pollution and negative environmental impacts. The economic activity has a significant impact on the environment since establishing the company with its location on a particular land area can significantly impact the environment. The technological process’s deployment is based on raw materials extracted from the environment to be processed into the finished product. As a result of these processes, different types of waste will enter the environment, influencing its quality. The manufacture of the product has a negative influence on the environment and the other phases of the enterprise’s economic activity; for example, the distribution involves the consumption of energy, which later turns into waste, affecting the environment.

Stage 2 (Determination of impacts exerted by economic activity on the environment). Environmental impact is the positive or negative effect that economic activity exercises or may have on the environment. The impact of economic activity on the environment is classified as direct and indirect. The direct impact is directly related to the production process. It can be characterized upstream or downstream of the production processes. Some forms of impact that directly influence the production activity of enterprises include demand for material resources necessary for production, the volume of emissions to air, water, and soil as a result of the production process, as well as waste unused. This step is essential in developing the accounting model because the environmental impact is the essential element to be integrated and reflected in the company’s managerial accounting system. Knowing the forms of the environmental impact exerted by economic activity contributes to the proper development of a model.

Stage 3 (Identifying and allocation of environmental costs caused by environmental impact). After determining the environmental impact, the monetary value of the enterprise’s environmental costs will be identified to reduce, prevent, or protect. Identifying environmental costs related to a product, work, service, and accounting recognition is required for correct management decisions. Knowing the environmental costs of current, future, and potential impact is the starting point for setting environmental spending cuts goals. Information on environmental costs is determined during the management process and is used to substantiate reliable production cost modeling decisions. To reflect environmental costs in accounting, we suggest analytical evidence [40] on types of specific environmental activities (sustainability and conservation expenses, expenses to neutralize the polluting activities of the various factors of production, measurement, and level of remediation control spending), or fields (air and climate protection, wastewater management, soil, and groundwater or surface water, the fight against noise and vibration, biodiversity protection, radiation protection). Identifying and adequately collecting physical and financial data will lead to improved decision making in the organization. 

Stage 4 (Internalization of externalities—the integration of environmental costs in production). For a long time, it was considered that the environment does not affect the production process. However, now that environmental impact analysis showed that economic activity affects the natural environment, it is essential to allocate environmental costs to each product, work, and service at each technological process stage. In the present, the prices include accounting information built as an expression of economic reality, without recognizing the impact on the environment [76]. The role of this model is to integrate environmental impact in accounting. The key element is the integration of these environmental externalities and their reflection in each company’s accounting. Externalities are consequences or effects that affect the life and activity of people and the natural environment. These represent costs or benefits and, although they occur, are not recognized as an expense or results obtained by the entity. Externalities are the gains or losses incurred either by individuals, resulting from actions initiated either by producers or consumers, or both, and for which no compensation is paid. Externalities are also called “third-party effects” or “neighborhood effects” [77]. Environmental damage by an enterprise and whose repair is the responsibility of the community is an externality. Environmental costs arise when the enterprise internalizes externalities, taking the responsibility to prevent or repair the damage caused. This stage’s main objective is the integration of environmental impact into accounting under environmental expenses and costs. Traditional accounting systems do not address this need, so the implementation of environmental accounting methods via specific environmental costing, for example, Target Costing, is a response to these concerns. 

Stage 5 (Identifying the environmental information provided by the environmental management accounting and their use for achieving environmental reporting or previsions). For businesses, environmental management accounting is designed to increase the efficiency and effects of environmental measures taken and to keep track of environmental expenses and income, report, and reflect them in the financial statements. The role of this step is to use environmental information provided by accounting for presentation and publication. The model offers a review of the allocation of environmental costs for each process or activity, conducting environmental reports by company-specific regulations. It can be said environmental management accounting measures the efficiency of environmental conservation activities, environmental performance index, and environmental efficiency of economic activities of companies, as part of financial performance associated with environmental measures. This step represents the accounting model’s finality for environmental coverage in accounting, so this information will be presented both internally and externally to meet managers, customers, suppliers, and investors’ needs. As financial statements provide monetary information, it is necessary to draw up annual environmental statements containing monetary, environmental information. This information is intended to present the real impact on the environment exerted by enterprise activity. The difficulty is the characteristics of the information must be presented in a rigorous, scientific, and technical form to allow experts to assess the level of pollution and be presented simply to be understood by non-specialist readers of the field.

## 6. Conclusions

Environmental management accounting helps to identify the environmental and production costs. Without its use, these costs will be ignored or underestimated. The correct identification of these costs allows the cost control, minimizing costs, and cleaner production techniques, thereby benefiting by reducing the organization’s production costs and improving environmental performance. Environmental management accounting also contributes to allocate the environmental costs to each product, process, or activity. It helps to correct the pricing of the product. Some products, those that are greater pollutants, may seem more profitable without the identification of costs on environmental protection. The purpose of environmental accounting is recognition and trying to find ways to mitigate the adverse effects of environmental activities and systems. Together with the benefits of environmental accounting, these objectives represent the strength for developing and implementing environmental accounting at the national, international, or global level.

Following the analysis of the questionnaire, the respondents consider that the integration of the environmental impact in accounting can be achieved by: i) identifying and evaluating, from a monetary perspective, the environmental impact (34.70%); ii) determining the environmental costs (24.30%); iii) integrating environmental costs in the decision-making process (23.60%); iv) through non-financial reporting (17.40%). Simultaneously, among the obstacles for implementing environmental accounting by the Romanian entities from the respondents’ perspective are the lack of an accounting model (48.60%) and the lack of a transparent application methodology (39.60%). At the same time, 140 respondents consider integrating environmental costs to substantiate the prices of products and services as a benefit that can generate the maximization of the entity’s results.

A hierarchy of environmental expenditures was made in terms of Romanian economic entities participating in the study: i) expenses for waste management (51.40%); ii) expenses for wastewater management (19.10%); iii) expenditure on air protection, expenditure on soil and groundwater protection, expenditure on the protection of biodiversity and landscape, expenditure on noise and vibration reduction, expenditure on radiation protection, expenditure on research and development in the field of environmental protection and other environmental activities (records a share of less than 10%).

The validation of hypotheses H1–H4 found that the CM-EMA model’s key point is to identify the environmental impact and quantify it as a monitoring of the environmental costs. CM-EMA places the product at the center of sustainable strategic management, following the products’ cost in correlation with the life cycle, especially the consumption of resources required by the design phase. Through this new approach of sustainable analysis of the production costs and the performances of the economic entities, the compact aims to integrate the four dimensions of sustainable management: the economic environment related to the market and competition, the integration of the competencies of the different functions; forecasting decisions regarding future results and close connection between planning and controlling current activities.

The CM-EMA model can be considered a step in establishing a possible framework for developing and implementing environmental accounting by Romania’s economic entities. The model is relevant in identifying environmental impacts, thus determining environmental costs, allocating the actual cost of each product, work, or service, and presenting environmental information to internal and external users, as an essential tool in integrating environmental impact in the accounting system of businesses.

The limit of the present research may be the small number of respondents to the questionnaire. As a future research direction, the practical test of the conceptual model CM-EMA applied initially only to an economic entity to validate its usefulness and identify the modalities of extension to different fields of activity.

## Figures and Tables

**Table 1:** Cost categories for EMA.

Types	Description
Materials Costs of Product Outputs	The purchase costs of natural resources such as water and other materials are converted into products, by-products, and packaging.
Materials Costs of Non-Product Outputs	The purchase (and sometimes processing) costs of energy, water, and other materials that become Non-Product Output (Waste and Emissions).
Waste and Emission Control Costs	Costs for: handling, treatment and disposal of Waste and Emissions; remediation and compensation costs related to environmental damage; and any control-related regulatory compliance costs.
Prevention and Other Environmental Management Costs	Costs of preventive environmental management activities such as cleaner production projects, costs for other environmental management activities such as environmental planning and systems, environmental measurement, environmental communication, and any other relevant activities.
Research and Development Costs	Costs for Research and Development projects related to environmental issues.
Less Tangible Costs	Includes both internal and external costs related to less tangible issues. Examples include liability, future regulations, productivity, company image, stakeholder relations, and externalities.

Source: International Federation of Accountants (IFAC), 2005.

**Table 2:** Descriptive analysis of the viability questionnaire.

Domains	No. of Item	Cronbach’s Alpha	Mean	Variance
EMAQ Total	32	0.970	2.791	1.706
Profile of the economic entity	10	0.967	3.150	.595
Social responsibility	13	0.873	2.276	1.911
Use of EMA	9	0.908	3.137	2.348

**Table 3:** Entities surveyed.

Sphere of Activity	Number of Entities Surveyed	%
Forestry, logging, and related services	35	12
Extractive industry	85	30
Manufacturing industry	41	14
Production and supply of electricity and heat, gas and water	30	10
Construction	15	5
Transportation	33	12
Public Administration	49	17
Total	288	100%
Chi2	0.370	
Significance level	*p* < 0.05	

**Table 4:** Assessing the dispersion of responses for each domain.

		Sale Price	Product Quality	Market Image	Bioproduct Characteristics	Involvement in Ecological Investments	Environmental Policies and Strategies	Target Market
N	Valid	288	288	288	288	288	288	288
	Missing	0	0	0	0	0	0	0
Mean		3.49	3.48	3.58	3.60	3.51	3.65	1.80
Std. Error of Mean		0.059	0.059	0.054	0.057	0.060	0.058	0.035
Std. Deviation		1.002	1.002	0.918	0.975	1.026	0.984	0.601

**Table 5:** Ranking of comparative economic entities against their main competitors.

Tool	1	2	3	4	5
Product selling price	10	35	90	110	43
Product quality	6	40	102	90	50
Market image of the products	2	32	98	108	48
Bio features of the products	1	44	78	110	55
Involvement in green investments	0	55	92	81	60
Environmental policies and strategies	0	42	82	100	64

**Table 6:** Level of implementation of international standards in environmental protection.

Tool	Known	Used	In Progress	I Do Not Know
ISO 14001 Environmental Management System	122 (42.40%)	101 (35.10%)	63 (21.90%)	2 (0.70%)
ISO 14031 Evaluation of environmental performance	130 (45.10%)	140 (48.60%)	10 (3.50%)	8 (2.80%)
ISO 9000 Quality system	128 (44.40%)	140 (48.60%)	14 (4.90%)	6 (2.10%)
Development of environmental policy	126 (43.80%)	144 (50%)	16 (5.60%)	2 (0.70%)
Development of environmental budgets	115 (39.90%)	150 (52.10%)	19 (6.60%)	4 (1.40%)
Reporting non-financial information in annual financial statements	108 (37.50%)	150 (52.10%)	22 (7.60%)	8 (2.80%)
Reporting non-financial information in other reports	100 (34.70%)	153 (53.10%)	24 (8.30%)	11 (3.80%)

**Table 7:** Integration of environmental impact, barriers in this process.

Integration of Environmental Impact	Frequency	Percent	Barriers in This Process	Frequency	Percent
Monetary identification and evaluation of the environmental impact	100	34.70	Lack of classification of environmental costs	8	2.80
Determination of environmental costs	70	24.30	Training in environmental accounting is at the beginning	26	9.00
Integration of environmental costs in the decision-making process	68	23.60	Lack of a conceptual accounting model	140	48.60
Reporting of non-financial information related to environmental impact	50	17.40	Lack of a clear methodology for integrating environmental impact	114	39.60

**Table 8:** Verification of Hypothesis 1.

		FA	O	CS	MT	Leg	B	Act	Ob	EI	EII
**Kendall’s tau_b**	Field of activity (FA)	1.000									
	Ownership (O)	0.691 **	1.000								
	Company size (CS)	0.841 **	0.731 **	1.000							
	Market targeted by the company (MT)	0.727 **	0.808 **	0.756 **	1.000						
	Legislation (Leg)	0.903 **	0.713 **	0.811 **	0.755 **	1.000					
	Beliefs (B)	0.449 **	0.283 **	0.384 **	0.421 **	0.452 **	1.000				
	Actions (Act)	0.927 **	0.706 **	0.837 **	0.763 **	0.900 **	.445 **	1.000			
	Obstacles (Ob)	0.888 **	0.737 **	0.852 **	0.787 **	0.888 **	0.459 **	0.930 **	1.000		
	Environmental Impact (EI)	0.219 **	0.123 *	0.178 **	0.293 **	0.226 **	0.167 **	0.239 **	0.253 **	1.000	
	Environmental Impact Integration (EII)	0.354 **	0.199 **	0.288 **	0.473 **	0.366 **	0.223 **	0.381 **	0.357 **	0.619 **	1.000

*. Correlation is significant at the 0.05 level (2-tailed). **. Correlation is significant at the 0.01 level (2-tailed).

**Table 9:** Cross-analysis of environmental impact variables * environmental management system.

		Cases									
		Valid			Missing				Total		
		N		Percent	N		Percent		N		Percent
The activity undertaken by the economic entity has any impact on the environment * The environmental management system		288		100.0%	0		0.0%		288		100.0%
		**Environmental management**								**Total**	
		**Known**	**In use**			**In progress**		**Not known**			
Environmental impact	Yes	122	101			37		0		260	
	No	0	0			26		2		28	
Total		122	101			63		2		288	

**Table 10:** Analysis of the symmetry of the variables environmental impact * environmental management system.

		Value	Approx. Sig.
Nominal by Nominal	Phi	0.629	0.000
	Cramer’s V	0.629	0.000
N of Valid Cases		288	

**Table 11:** Person Correlation.

		EI	EMS	EPM	SC	DPM	EBM	RNFrf	RNFar
Pearson Correlation	EI	1.000							
	EMS	0.911	1.000						
	EPM	0.845	0.844	1.000					
	SC	0.859	0.861	0.977	1.000				
	DPM	0.879	0.884	0.964	0.970	1.000			
	EBM	0.685	0.742	0.826	0.829	0.852	1.000		
	RNFrf	0.494	0.594	0.678	0.679	0.696	0.633	1.000	
	RNFar	0.356	0.493	0.580	0.579	0.590	0.527	0.442	1.000
Sig. (1-tailed)	EI	.	0.000	0.000	0.000	0.000	0.000	0.000	0.000
	EMS	0.000	.	0.000	0.000	0.000	0.000	0.000	0.000
	EPM	0.000	0.000	.	0.000	0.000	0.000	0.000	0.000
	SC	0.000	0.000	0.000	.	0.000	0.000	0.000	0.000
	DPM	0.000	0.000	0.000	0.000	.	0.000	0.000	0.000
	EBM	0.000	0.000	0.000	0.000	0.000	.	0.000	0.000
	RNFrf	0.000	0.000	0.000	0.000	0.000	0.000	.	0.000
	RNFar	0.000	0.000	0.000	0.000	0.000	0.000	0.000	.
N		288	288	288	288	288	288	288	288

**Table 12:** Correlations of the analyzed variables.

		The Extent to Which the Economic Activity Generates Information on Environmental Costs	The Annual Value of the Environmental Costs	Weight in the Total of the Expenses Recorded by the Economic Entity
The extent to which the economic activity generates information on environmental costs	Pearson Correlation	1	0.870 **	0.427 **
	Sig. (2-tailed)		0.000	0.000
	N	288	288	288
The annual value of the environmental costs	Pearson Correlation	0.870 **	1	0.435 **
	Sig. (2-tailed)	0.000		0.000
	N	288	288	288
Weight in the total of the expenses recorded by the economic entity	Pearson Correlation	0.427 **	0.435 **	1
	Sig. (2-tailed)	0.000	0.000	
	N	288	288	288

**. Correlation is significant at the 0.01 level (2-tailed).

**Table 13:** The result of the cross-analysis of the variables Benefits of integrating the environment in the accounting system * Decision of integrating the environmental costs in the prices of the products.

		Cases						
		Valid			Missing		Total	
		N	Percent		N	Percent	N	Percent
Integration of the environment in the accounting system has been a benefit * In the strategy of pricing, the environmental costs influence the decision		288	100.0%		0	0.0%	288	100.0%
		In the pricing strategy, environmental costs influence the decision						Total
		To a large extent		If we have information about environmental costs, we take them into account, but not strictly		We do not take environmental costs into account when making product pricing decisions		
The integration of the environment into the accounting system was a benefit	Yes	140		100		0		240
	No	0		26		0		26
	Do not know	0		14		8		22
Total		140		140		8		288

**Table 14:** Spearman’s rank correlation for the variable environmental benefits in the accounting system * decision to integrate environmental costs into the product prices.

			The Integration of the Environment into the Accounting System Was a Benefit	In the Pricing Strategy, Environmental Costs Influence the Decision
Spearman’s rho	The integration of the environment into the accounting system was a benefit	Correlation Coefficient	1.000	0.485 **
		Sig. (2-tailed)	.	0.000
		N	288	288
	In the pricing strategy, environmental costs influence the decision	Correlation Coefficient	0.485 **	1.000
		Sig. (2-tailed)	0.000	.
		N	288	288

**. Correlation is significant at the 0.01 level (2-tailed).

**Table 15:** Correlation Matrix.

		Integrated Environmental Impact	Barriers Integrated Environmental Impact	Generate Environmental Cost	Environmental Expenses Recorded	Annual Value	Share in Total Expenses	Environmental Cost Strategy	Optimal Method	Attitude Top Management
Correlation	Integrated environmental impact	1.000	0.436	0.280	0.449	0.490	0.888	0.425	0.431	0.415
	Barriers integrated environmental impact	0.436	1.000	0.812	0.859	0.911	0.387	0.839	0.490	0.825
	Generate environmental cost	0.280	0.812	1.000	0.842	0.921	0.249	0.715	0.672	0.791
	Environmental expenses recorded	0.449	0.859	0.842	1.000	0.914	0.399	0.702	0.551	0.867
	Annual value	0.490	0.911	0.921	0.914	1.000	0.435	0.806	0.644	0.852
	Share in total expenses	0.888	0.387	0.249	0.399	0.435	1.000	0.422	0.480	0.368
	Environmental cost strategy	0.425	0.839	0.715	0.702	0.806	0.422	1.000	0.550	0.681
	Optimal method	0.431	0.490	0.672	0.551	0.644	0.480	0.550	1.000	0.414
	Attitude top management	0.415	0.825	0.791	0.867	0.852	0.368	0.681	0.414	1.000
	Sig. (1-tailed)	0.000		0.000	0.000	0.000	0.000	0.000	0.000	0.000
